# Mobile Phone Access and Willingness Among Mothers to Receive a Text-Based mHealth Intervention to Improve Prenatal Care in Northwest Ethiopia: Cross-Sectional Study

**DOI:** 10.2196/pediatrics.9618

**Published:** 2018-10-17

**Authors:** Berhanu Endehabtu, Adane Weldeab, Martin Were, Richard Lester, Abebaw Worku, Binyam Tilahun

**Affiliations:** 1 eHealthLab Ethiopia Department of Health Informatics University of Gondar Gondar Ethiopia; 2 Health Education and Behavioral Sciences Unit University of Gondar Gondar Ethiopia; 3 Department of Biomedical Informatics Vanderbilt University Vanderbilt, IN United States; 4 Division of infectious disease Faculty of Medicine University of the British Columbia Vancouver, BC Canada; 5 Institute of Public Health University of Gondar Gondar Ethiopia

**Keywords:** mHealth, mobile phone, pregnant women, SMS, willingness, Ethiopia, antenatal care, maternal health

## Abstract

**Background:**

Maternal mortality remains high in many low- and middle-income countries where limited access to health services is linked to low antenatal care utilization. Effective communication and engagement with care providers are vital for the delivery and receipt of sufficient health care services. There is strong evidence that simple text-based interventions can improve the prenatal care utilization, but most mobile health (mHealth) interventions are not implemented on a larger scale owing to the lack of context and preliminary evidence on how to make the transition.

**Objective:**

The objective of this study was to determine access to mobile phones by pregnant women attending antenatal care as well as willingness to receive a text message (short message service, SMS)–based mHealth intervention for antenatal care services and identify its associated factors among pregnant women attending an antenatal care clinic in Gondar Town Administration, Northwest Ethiopia, Africa.

**Methods:**

A cross-sectional quantitative study was conducted among 422 pregnant women attending antenatal care from March 27 to April 28, 2017. Data were collected using structured questionnaires. Data entry and analysis were performed using Epi-Info version 7 and SPSS version 20, respectively. In addition, descriptive statistics and bivariable and multivariable logistic regression analyses were performed. Furthermore, odds ratio with 95% CI was used to identify factors associated with the willingness to receive a text message–based mHealth intervention.

**Results:**

A total of 416 respondents (response rate 98.6%, 416/422) were included in the analysis. About 76.7% (319/416) of respondents owned a mobile phone and 71.2% (296/416) were willing to receive an SMS text message. Among the mobile phone owners, only 37.6% (120/319) were having smartphones. Of all women with mobile phones, 89.7% (286/319) described that they are the primary holders of these phones and among them, 85.0% (271/319) reported having had the same phone number for more than a year. Among the phone owners, 90.0% (287/319) described that they could read and 86.8% (277/416) could send SMS text messages using their mobile phones in their day-to-day activities. Among pregnant women who were willing to receive SMS text messages, about 96.3% (285/296) were willing to receive information regarding activities or things to avoid during pregnancy. Factors associated with willingness were youth age group (adjusted odds ratio [AOR] 2.869, 95% CI 1.451-5.651), having attained secondary and higher educational level (AOR 4.995, 95% CI 1.489-14.773), and the frequency of mobile phone use (AOR 0.319, 95% CI 0.141-0.718).

**Conclusions:**

A high proportion of pregnant women in an antenatal care clinic in this remote setting have a mobile phone and are willing to receive an SMS text message–based mHealth intervention. Age, educational status, and the frequency of mobile phone use are significantly associated with the willingness to receive SMS text message–based mHealth interventions.

## Introduction

Maternal health is a critical issue to be addressed worldwide. Every minute of every day, somewhere in the world, women lose their lives because of complications related to pregnancy and childbirth. It is estimated that globally every day about 830 women die from preventable causes related to pregnancy and delivery, and about 99% of all maternal deaths occur in developing countries where access to antenatal care (ANC) services is limited [1]. A majority of these deaths are preventable through focused ANC service [2,3]. It has been widely stated that routine ANC visit is one of the most effective ways to reduce maternal morbidity and mortality [4]. Sub-Saharan Africa is the region with a high maternal mortality ratio and low ANC utilization [3].

According to the Ethiopian Demographic and Health survey 2016 key indicators report, maternal mortality ratio in Ethiopia is 412/100,000 [5]. Despite calls by the World Health Organization for a minimum of 4 ANC visits, only 32% of pregnant women in Ethiopia attend the recommended ANC service, with residential variations [5]. Urban women were more likely than rural women to have received ANC from a skilled provider. Among different barriers, limited access to health services and lack of effective communication are among the major barriers for low ANC [6]. A missed appointment is a major cause of inefficiency in health care delivery, with substantial financial costs for the health system, leading to delays in the diagnosis and appropriate treatment [7]. In addition, effective communication is vital for the delivery and receipt of sufficient health care services [8,9]. Hence, new and innovative strategies are required to overcome the problem and improve ANC services. The use of mobile telecommunication technology at medical and public health practice is gaining attention because it gives the chances to rapidly connect people, reducing delay across the chain of health decisions, and positively affecting the lives of many in the underserved population [10,11].

Mobile phones are increasingly used in health care systems in low- and middle-income countries and are considered as innovative solutions that have immense potential to overcome barriers of access to ANC service [12,13]. Several scientific papers have shown that short message service (SMS) text messaging-based mobile health (mHealth) intervention could potentially improve maternal and child health [13-18]. SMS-based mHealth messaging plays an important role in maternal health care like reminders for health care appointments [19].

In Ethiopia, mobile subscribers are increasing exponentially and the mobile network coverage is expanding [20]. Even though there is only one government-owned telecom company that provides the service, the use of phone call and SMS text messaging is expectantly increasing among health care workers in day-to-day health service provisions. There is a great opportunity to link the constantly growing mobile telecommunication technology with the many-sided maternal and child health care programs and strategies. Mobile phone ownership among pregnant women attending ANC and the willingness of women to receive SMS text messages remain unknown in the Ethiopian context. Before implementing the SMS text message–based mHealth intervention, it is essential to know the level of access to mobile phone and the willingness of women to receive SMS text message–based interventions.

Therefore, the objectives of this study are as follows:

- To assess the access to mobile phone among pregnant women attending ANC clinics in Gondar Town Administration
- To determine the willingness of those women to receive SMS text message–based mHealth interventions
- To identify the factors associated with the willingness to receive SMS text message–based mHealth interventions among pregnant women attending ANC

## Methods

### Study Design and Setting

A cross-sectional quantitative study was conducted at 8 health facilities from March 27 to April 28, 2017, in the Gondar Town Administration, Northwest Ethiopia. The Gondar Town Administration is divided into 8 clusters namely Gondar, Ginbot 20, Azezo, Gebriel, Maraki, Woleka, Teda, and Belajig; the administration has a total of 24 Kebele (13 urban and 11 rural). In addition, the administration has a total of 23 public health facilities, 1 referral hospital, 8 health centers, and 14 health posts. Of the estimated population of the town, 49.5% (162,192/327,661) are females and 50.5% (165,469/327,661) are males. Among the total population, 260,183 are urban inhabitants and the rest 67,478 are rural inhabitants. In the 2016-17 budget year, the number of women in the reproductive age group was 77,262 and the estimated number of pregnancies was 11,042 (data from Gondar Town health department). In the Ethiopian context, health center means a health facility that provides primary health care and urban area implies a town that consists of at least 2000 residences.

### Study Subjects

All women who were pregnant and attending ANC service at health centers during the study period were used as the study population.

### Sample Size

The sample size of this study was determined using the single population proportion formula (*n*=(*z* α/2)^2^pq/∂^2^) with the following assumptions:

*n*=the required sample size; z=the value of the standard normal distribution corresponding to α/2=1.96;
*p*=the proportion of pregnant women who are attending ANC and willing to be contacted by mobile phone;
*q*=1-
*p*, the proportion of pregnant women who are attending ANC and not willing to be contacted by mobile phone; and ∂= margin of error 5% (0.05).

We could not find any studies conducted to determine the mobile phone ownership among pregnant women attending ANC in Ethiopia, although the general subscriber identity module (SIM or subscriber identification module) population in Ethiopia is 48.3% [20]. Moreover, we could not find any study conducted in Ethiopia to determine the willingness of pregnant women who are attending ANC to receive SMS text message–based mHealth interventions for ANC services. Therefore, we assumed that 50% of pregnant women are willing to receive an SMS text message–based mHealth intervention for ANC services. The maximum sample size was 384 using the proportion of pregnant women who were attending ANC and willing to be contacted by mobile phone. Considering a 10% nonresponse rate, we calculated the final sample size to be 422. Thus, a systematic random sampling technique was performed to select 422 study participants.

### Data Collection Tools and Procedure

Women exiting ANC visit were approached for interviews at each of the 8 health centers. The interviews included sociodemographic characteristics, physical accessibility to a health care facility, electricity and network availability, patterns of mobile phone use, and women’s opinion and willingness to receive health information via SMS text messages through mobile phones. Questionnaires were first developed in English, which then underwent forward and backward translation to ensure semantic consistency (English to Amharic then English), for the appropriateness and easiness in approaching study participants. Of note, a pretest of the questionnaire was conducted among pregnant women attending ANC (5% of the sample) before the study period at health centers in the Debre-tabor Town Administration, following which necessary modifications were made on the basis of pretest findings. Research personnel, including 2 health information technicians, 2 nurses with bachelor degrees acting as supervisors, and 8 clinical nurses serving as data collectors or interviewers, received a 1-day training course on implementing the evaluation, which included training on research ethics, providing informed consent, data collection procedures, data collecting tools, how to approach participants, data confidentiality, respondents’ right and all the study protocols to be followed throughout the course of the data collection period. In addition, continuous monitoring by supervisors was done throughout the data collection period to ensure that the data were collected according to the study protocol. The completed questionnaires were stored in binders in nurses’ class until collected by the principal investigator.

### Data Management and Statistical Analysis

Data were entered using Epi-Info version 7 and transferred to SPSS version 20. Descriptive statistics were performed to describe the study population. We used the binary logistic regression to analyze the association of each study variable on the outcome variable. The dependent variable was designated as “no”=0 (have no willingness) and “yes”=1 (for having willingness). Variables significantly associated with the outcome variable (*P*<.2) in the bivariable analysis were included in the multivariable logistic regression analysis for controlling the possible effects of confounders. In the multivariable analysis, Hosmer and Lemeshow goodness-of-fit test was performed (*P*=.76), and variables which were significant based on the adjusted odds ratio (AOR), with 95% CI and *P*<.05, were considered to be the determinant factors of willingness to receive an SMS text message–based mHealth intervention.

### Ethical Consideration

Ethical clearance was obtained from the ethical review board of the University of Gondar. In addition, oral consent was obtained from study participants after narrating the objective of the study; they were also informed about the benefits of the study. If they felt discomfort during the interview, they were informed that they could stop at any time. Moreover, confidentiality assurance was provided to study participants on any information provided by them; the data collection procedure was anonymous, and their privacy was upheld.

## Results

### Mobile Phone Ownership

A total of 422 pregnant women from 8 public health centers were approached; of them, 416 responded to complete all the questionnaires at each health center (response rate 98.6%). Of all respondents, 81.3% (338/416) were urban residents. The age of respondents ranged from 18 to 45 years (mean age 26.6 [SD 5.4] years). In addition, 94.5% (393/416) of them were married, 67.3% (280/416) were housewives, and 51.7% (215/416) had attained at least secondary educational level (Table 1).

Table 1 shows that 76.7% (319/416) pregnant women owned a mobile phone; of them, 63.0% (201/319) were in the age group of ≥25 years. Almost all mobile phone owners were married (303/319, 95.0%) and 63.0% (201/319) had attained secondary and higher educational level.

### Patterns of Mobile Phone Use

In this study, 97 pregnant women had no mobile phone; main reasons reported for not owning a mobile phone currently were cannot afford to buy (53/97, 54.6%), followed by mobile phone broken (17/97, 17.5%).

Of all women with mobile phones, 89.7% (286/319) described that they are the primary holders of these phones; however, 29.8% (94/319) of them described that they share their mobile phone with others, especially with other family members. In addition, 51.4% (164/319) of them locked their mobile phone with a password and 30.1% (96/319) put their mobile phone in a place where others can see and access it easily. Furthermore, 31.3% (100/319) of women reported that there were times or places where they did not answer calls and 14.7% (47/319) reported switching-off mobile phones during the daytime.

Of the respondents with mobile phones, 85.0% (271/319) reported having had the same phone number for more than a year; the other 15.0% (48/319) reported changing their mobile phone number in the last 1 year. In addition, 37.6% (120/319) of current mobile phone owners had smartphones. Among current mobile phone owners, 47.3% (151/319) of pregnant women described that they accessed the internet through their mobile phones, which could be either a basic phone or smartphone; of them, 94.7% (143/151) reported using the Facebook app mainly to stay in touch with friends and relatives through this social media platform.

**Table 1:** Sociodemographic characteristics of pregnant women attending antenatal care follow-up at health centers in the Gondar Town Administration, Northwest Ethiopia, 2017.

Sociodemographic characteristics		Pregnant women (n=416), n (%)		Pregnant women owning a mobile phone (n=319), n (%)	
**Age**					
	15-24		160 (38.5)		118 (37.0)
	≥25		256 (61.5)		201 (63.0)
**Residence**					
	Urban		338 (81.3)		298 (93.4)
	Rural		78 (18.7)		21 (26.6)
**Marital status**					
	Not married		14 (3.4)		10 (3.1)
	Married		393 (94.5)		303 (95)
	Other^a^		9 (2.2)		6 (1.9)
**Educational status**					
	Cannot read and write		70 (21.9)		21 (6.6)
	Informal education		21 (5.0)		12 (3.8)
	Primary		110 (26.4)		85 (26.6)
	Secondary and above		215 (51.7)		201 (63.0)
**Occupation**					
	Housewife		280 (67.3)		192 (60.2)
	Civil servant		49 (11.8)		49 (15.4)
	Merchant		48 (11.5)		46 (14.4)
	Daily laborer		24 (5.8)		20 (6.3)
	Student		12 (2.9)		10 (3.1)
	Other^b^		3 (0.7)		2 (0.6)
**Who do you live with**					
	Family		385 (92.5)		294 (92.2)
	Alone		24 (5.8)		21 (6.6)
	Parents		7 (1.7)		4 (1.3)
**Number of children**					
	No child		185 (44.5)		160 (50.2)
	1		77 (18.5)		59 (18.5)
	2		80 (19.2)		60 (18.8)
	3		44 (10.6)		29 (9.1)
	≥4		30 (7.2)		11 (3.4)

^a^Separated, windowed, and died.

^b^Farmer, driver, and jobless.

Of all respondents with mobile phones, 90.0% (287/319) described that they could read and 86.8% (277/416) could send SMS text messages using their mobile phones. However, 6.3% (18/319) of them described that they deleted SMS text messages without reading them. Among those who currently owned a mobile phone, only 33.2% (106/319) used their mobile phone for health-related information or purposes; of them, 50.0% (53/106) respondents used to set the alarm to take medication, 36.9% (39/106) received health-related SMS text messages or calls from health organization or health care providers, 32.1% (34/106) used their phones to consult health professionals, and 24.5% (26/106) used their phones to browse health-related information using the internet.

### Willingness to Receive a Short Message Service Text Message–Based mHealth Intervention

As shown in Table 2, those who owned smartphones were more willing to receive SMS text messages than those who owned standard feature phones (101, 84.2% vs 144, 72.4%). Respondents who had used mobile phones to send SMS text messages were also more willing to receive SMS text message–based health interventions compared with those who never used the SMS text messaging service before (80.3% vs 67.8%).

Respondents who used their phones as an alarm reminder to take their medication were more willing to receive SMS text message–based mHealth interventions than those who did not (85% vs 75.2%). This willingness was also observed in respondents who received SMS text messages from health organization before compared with those who had not (79.5% vs 76.4%). The frequency of the mobile phone use also correlated with the willingness to accept an SMS text message–based mHealth intervention. Of note, 229 women (80.1%) who “always” used their mobile phones were willing to receive SMS text messages compared with those who only used their mobile phones “sometimes” 16 (48.5%). The willingness to receive SMS text messages was higher among respondents who locked their mobile phone with a password than those who did not lock their mobile phone with a password (80.5% vs 72.9%). In addition, internet users via their mobile phone were also more willing than noninternet users (82.1% vs 72%).

### Attitude and Willingness to Receive Short Message Service Text Message–Based mHealth Interventions

In this study, 71.2% (296/416) respondents were willing to receive SMS text messages with information regarding ANC (Table 3). The pregnancy period at which they would want to begin receiving SMS text messages varied greatly: from 1 month, 117/296, 39.5%; from 3 months, 163/296, 55.1%; and from 6 months, 10/296, 3.4%, and at 9 months of pregnancy but before delivery 2% (6/296).

The time of day at which they would want to receive SMS text messages varied greatly. Overall, 19.9% (59/296) of women preferred receiving an SMS text message at morning only (8 am-before 12 pm), 6.8% (20/296) in the afternoon only (12 pm-before 4 pm), 12.8% (38/296) in the evening only (4 pm-before 8 pm), whereas 60.5% (179/296) described they could receive the SMS text messages at any time of the day. Among respondents who were willing to receive SMS text messages, more than three-fourth preferred receiving them at a frequency of once a week.

Overall, women were interested in receiving pregnancy and related information via SMS text messages. Among pregnant women who were willing to receive SMS text messages, about 96.3% (285/296) were willing to receive information regarding activities or things to avoid during pregnancy.

Those who intended to receive health information regarding delivery courses via SMS text messages were 90.5% (268/296). In addition, respondents were willing to receive SMS text messages about what to expect at various stages of pregnancy (249/296, 84.1%), prenatal dietary information (236/296, 79.7%), appointment reminders (209/296, 70.6%), when to call a doctor during pregnancy (107/296, 36.1%), and physical activities during pregnancy (88/296, 29.7%). Among respondents who were willing to receive SMS text messages, 78.4% (232/296) indicated that they were willing to pay for the service based on the current SMS text messaging rates.

### Factors Associated With the Willingness to Receive Short Message Service Text Messages

Variables in the bivariable analysis of sociodemographics, patterns of mobile phone use, access to a health facility, and ANC-related factors around the willingness to receive SMS text messages that had *P*<.20 were further considered in the multivariable analysis (Table 4). The multivariable logistic regression analysis revealed that the following factors were significantly associated with the willingness to receive SMS text message–based mHealth intervention among pregnant women: youth age group (15-24 years, *P*=.002); educational status (primary: *P*=.05; secondary and above: *P*=.004); and mobile phone use as “always” (*P*=.006; Table 4). Respondents in the youth age group were 2.87 times (AOR 2.869, 95% CI 1.451-5.651) more likely willing to receive SMS text message–based mHealth interventions than those aged >25 years. Respondents with secondary and higher educational level were 5 times (AOR 4.995, 95% CI 1.689-14.773) more willing to receive SMS text messages than those with educational level below secondary. Respondents who used a mobile phone “sometimes” were 68.1% (AOR 0.319, 95% CI 0.141-0.718) less likely to be willing than those who used mobile phone always.

**Table 2:** Willingness to receive the short message service (SMS) text message–based mHealth intervention by patterns of mobile phone use among pregnant women attending antenatal care follow-up at health centers in the Gondar Town Administration, Northwest Ethiopia, 2017 (N=319).

Mobile phone use patterns		Total, n (%)	Willingness to receive SMS text messages, n (%)	
			Yes	No
**Mobile phone type**				
	Smart	120 (37.6)	101 (84.2)	19 (15.8)
	Standard	199 (62.4)	144 (72.4)	55 (27.6)
**Sent an SMS text message via mobile phone before**				
	Yes	229 (71.8)	184 (80.3)	45 (19.7)
	No	90 (28.2)	61 (67.8)	29 (32.2)
**Used mobile phone for health information before**				
	Yes	106 (33.2)	85 (80.2)	21 (19.8)
	No	213 (66.8)	160 (75.1)	53 (24.9)
**Used mobile phone to set alarm for taking medication**				
	Yes	53 (16.6)	45 (85)	8 (15)
	No	266 (83.4)	200 (75.2)	66 (24.8)
**Received SMS text messages from health organization before**				
	Yes	39 (12.2)	31 (79.5)	8 (19.5)
	No	280 (87.8)	214 (76.4)	66 (23.6)
**Consulted health care professionals**				
	Yes	34 (10.7)	26 (76.5)	8 (23.5)
	No	285 (89.3)	219 (77)	66 (23)
**Used the internet to browse health-related data**				
	Yes	26 (8.2)	20 (77)	6 (23)
	No	293 (91.9)	225 (76.8)	68 (23.8)
**Frequency of mobile phone use**				
	Always	286 (89.6)	229 (80.1)	57 (19.9)
	Sometimes	33 (10.3)	16 (48.5)	17 (51.5)
**Changed your subscriber identity module (SIM) card in the last 12 months**				
	Yes	48 (15)	35 (72.9)	13 (27.1)
	No	271 (85)	210 (77.5)	61 (22.5)
**Have an additional SIM card**				
	Yes	22 (6.9)	20 (90.9)	2 (9.1)
	No	297 (93.1)	225 (75.8)	72 (24.2)
**Switch off your mobile phone during daytime**				
	Yes	47 (14.7)	34 (72.3)	13 (27.7)
	No	272 (85.3)	211 (77.6)	61 (22.4)
**There are times or places when calls are not answered**				
	Yes	108 (33.9)	82 (75.9)	26 (24.1)
	No	211 (66.1)	163 (77.5)	48 (22.7)
**There are times, places, or situations when unknown calls are unanswered**				
	Yes	100 (31.3)	75 (75)	25 (25)
	No	219 (68.7)	170 (77.6)	49 (22.4)
**Locked the mobile phone with a password**				
	Yes	164 (51.4)	132 (80.5)	32 (19.5)
	No	155 (48.9)	113 (72.9)	42 (27.1)
	No	223 (69.9)	182 (81.6)	41 (18.4)
**Shared the mobile phone with others in the house**				
	Yes	95 (29.8)	64 (67.4)	31 (32.4)
	No	224 (70.2)	181 (80.8)	43 (19.2)
**Can send an SMS text message**				
	Yes	277 (86.8)	219 (79.1)	59 (209)
	No	42 (13.2)	26 (61.9)	16 (38.1)
**Can read an SMS text message**				
	Yes	287 (90)	226 (78.7)	61 (21.3)
	No	32 (10)	19 (59.4)	13 (40.6)
**Deleted an SMS text message without reading**				
	Yes	18 (6.3)	17 (94.4)	1 (5.6)
	No	269 (93.7)	211 (78.4)	58 (21.6)
**Likelihood of an SMS text message to be seen by others**				
	Very likely	26 (8.1)	17 (65.4)	9 (34.6)
	Likely	57 (17.9)	38 (66.7)	19 (33.3)
	Unlikely	71 (22.3)	55 (77.5)	16 (22.3)
	Very unlikely	165 (51.7)	135 (81.8)	30 (18.2)
**Used the internet via your mobile phone**				
	Yes	151 (47.3)	124 (82.1)	27 (17.9)
	No	168 (52.7)	121 (72)	47 (28)
**Chatted with friends and relatives**				
	Yes	143 (94.7)	123 (86)	20 (14)
	No	8 (5.3)	1 (12.5)	7 (87.5)
**Used the mobile phone to send emails**				
	Yes	88 (58.3)	77 (87.5)	11 (12.5)
	No	63 (41.7)	47 (74.6)	16 (25.4)
**Used the mobile phone to browse information**				
	Yes	46 (30.5)	39 (84.8)	7 (15.2)
	No	105 (69.5)	85 (81)	20 (19))
**Used the mobile phone for entertainment**				
	Yes	48 (31.8)	42 (87.5)	6 (12.5)
	No	103 (68.2)	82 (79.6)	21 (20.4)

**Table 3:** Attitude and willingness to receive short message service (SMS) text message–based mHealth interventions among pregnant women attending antenatal care follow-up at health centers in the Gondar Town Administration, Northwest Ethiopia, 2017.

Attitude and willingness to receive SMS text message			Pregnant women, n (%)
**Willing to receive SMS text messages (n=416)**			
	Yes		296 (71.2)
	No		120 (28.8)
**Reason not to be willing (n=120)**			
	Ruins privacy		50 (41.7)
	SMS text message is annoying		2 (1.6)
	Difficult to operate		54 (45)
	Not important		14 (11.7)
**Preferred time to begin receiving SMS text messages (n=296)**			
	Before 1 month		117 (39.5)
	From 3 months		163 (55.1)
	From 6 months		10 (3.4)
	From 9 months (before birth		6 (2.0)
**Preferred time of the day for receiving SMS text messages (n=296)**			
	Morning (8 am-before 12 pm)		59 (19.9)
	Afternoon (12 pm-before 4 pm)		20 (6.8)
	Evening (4 pm-before 8 pm)		38 (12.8)
	Any time		179 (60.5)
**Preferred frequency (n=296)**			
	1 per week		223 (75.3)
	3 per week		70 (23.6)
	5 per week		2 (0.7)
	7 per week		1 (0.3)
**Will pay for the service (n=296)**			
	Yes		232 (78.4)
	No		64 (21.6)
**Preferred pregnancy information to receive (n=296)**			
	**Activities or things to avoid**		
		Yes	285 (96.3)
		No	11 (3.7)
	**When to call a doctor**		
		Yes	107 (36.1)
		No	189 (63.9)
	**Diet**		
		Yes	236 (79.7)
		No	60 (20.3)
	**Appointment reminders**		
		Yes	209 (70.6)
		No	87 (29.4)
	**What to expect at various stages of pregnancy**		
		Yes	249 (84.1)
		No	47 (15.9)
	**Physical activity**		
		Yes	88 (29.7)
		No	208 (70.3)
	**Pregnancy and delivery courses**		
		Yes	268 (90.5)
		No	28 (9.5)

**Table 4:** Bivariable and multivariable analyses of factors with the willingness to receive short message service (SMS) text message–based mHealth interventions to improve antenatal care (ANC) among pregnant women attending ANC at health centers in the Gondar Town Administration, Northwest Ethiopia (N=416).

Factors		Willingness, n		Crude odds ratio (95% CI)	Adjusted odds ratio (95% CI)
		Yes	No		
**Age (years)**					
	15-24	133	27	2.810 (1.729-4.569)	2.869 (1.451-5.651)^a^
	≥25	163	93	Ref^b^	Ref
**Place of residence**					
	Urban	255	83	2.773 (1.667-4.612)	—
	Rural	41	37	Ref	—
**Educational level**					
	Cannot read and write	34	36	Ref	Ref
	Informal education	13	8	1.721 (0.634-4.666)	5.032 (0.792-31.978)
	Primary	78	32	2.581 (1.383-4.815)	3.040 (1.001-9.230)^a^
	Secondary and above	171	44	4.115 (2.318-7.305)	4.995 (1.489-14.773)^a^
**Type of mobile phone^c^**					
	Smart	101	19	2.030 (1.136-3.627)	—
	Standard	144	55	Ref	—
**Frequency of mobile phone use^c^**					
	Always	229	57	Ref	Ref
	Some times	16	17	0.234 (0.112-0.492)	0.319 (0.141-0.718)^a^
**Lock mobile phone with a password^c^**					
	Yes	132	32	0.110 (0.652-1.102)	—
**Share mobile phone with others in the house**					
	Yes	64	31	Ref	—
	No	181	43	2.039 (1.181-3.508)	—
**Use internet^c^**					
	Yes	124	27	1.784 (1.044-3.047)	—
	No	121	47	Ref	Ref

^a^Statistically significant at *P*<.05.

^b^Ref: reference.

^c^Participants with mobile phone ownership, n=319.

## Discussion

### Principal Findings

This study shows that access to mobile phone among pregnant women attending ANC at health centers in the Gondar Town Administration was high, with over three-quarters of women owning phones in this study. In addition, age, educational level, and frequency of the mobile phone use were among the notable factors associated with the willingness of pregnant women to receive SMS text message–based mHealth interventions.

The mobile phone ownership of women in this study (319/416, 76.7%) is lower than that reported in studies from Argentina (93.2%) [19] and South Africa (84%) [21]; this discrepancy could be attributed to the difference in the information and communication technology infrastructure and the socioeconomic status among countries. In addition, this is lower than studies conducted in Nigeria among women attending a tertiary facility for childhood immunization (99%); this disparity could be attributed to the study setting—a tertiary hospital—which mostly serves urban residents. Another possible explanation could be the difference in the information communication development index [22]. The mobile phone ownership in this study is nearly similar with that reported by a study conducted in Kenya (74.3%) [23]; it is also nearly similar with that reported by a study conducted in Ethiopia among antiretroviral therapy (ART) patients (76.1%) [24]. The possible explanation for this could be the similarity between study areas. This study found that the mobile phone ownership is much higher than the Ethiopian general mobile (SIM) population, which was reported to be only 48% [20]; this difference could be attributed to the study setting, which was one of the major towns in Ethiopia. In this study, most respondents were urban residents who had better access to telecommunication services. Hence, the findings of this study might not be generalizable to other areas of the country, especially in the rural communities.

In addition, 90.0% (287/319) and 86.8% (277/416) of current mobile phone owners could read and send an SMS text message, respectively, making an SMS text message–based intervention technically feasible. Furthermore, there is evidence that respondents were willing to receive SMS text message–based interventions, with a majority wanting the messages to begin early during pregnancy at 3 months, and a preference for once-weekly messages. Similar high willingness rates for SMS text message–based interventions have been observed in Argentina (96%) [19], South Africa (under option B+, 88.1%) [25], and Kenya (92%) [23]. Moreover, there is evidence of health-seeking behavior by pregnant women who wanted to receive information on what to avoid during pregnancy, diet, and information on pregnancy and delivery course

From those who were willing to receive SMS text messages, about 21.6% (64/296) of respondents were not willing to pay for SMS text messaging on current tele tariff rates, even though the benefit was clearly stated. An explanation for this could be that ANC services in Ethiopia are provided free of charge by the Ethiopian government and, thus, mothers might not want to take up any new costs. This finding has important implications for program managers and designers, as they may need to devise alternative payment mechanisms for SMS text messages in future intervention strategies.

This study identified some factors significantly associated with the willingness to receive SMS text message–based mHealth interventions among pregnant women. Younger pregnant women were more likely to be willing to receive SMS text messages; this result is consistent with a study from Kenya [26] and a study conducted in Ethiopia among ART patients [24].

This analysis indicated that women who achieved secondary or higher education were more likely to be willing to receive SMS text message–based mHealth interventions. This study is in line with a study from Ethiopia among ART patients [24] and Nigeria among women willingness for child immunization [27]. The result suggests that implementing the SMS text message–based mHealth intervention is particularly more feasible in the younger age group and more educated ones. However, it might be a potential drawback to implementing the SMS text message–based mHealth intervention program because about 21.6% (64/296) of respondents have no primary schooling; this highlights the influence of maternal educational status on the ANC utilization, as confirmed by other evidence [5]. Furthermore, it implies that before implementing the SMS text message–based mHealth intervention to improve the prenatal care utilization, apart from considering access to mobile phones, barriers that are related to the socioeconomic conditions of end users (especially the educational status) need to be fully explored and addressed.

This study shows that the place of residence, source of information for ANC, using the internet through mobile phones, mobile phone type, and mobile phone usage privacy variables, like locking the mobile phone with a password, and sharing a mobile phone with others were not found to be markedly associated with the willingness.

### Limitations

There are some limitations to this study. As the study was an institution–based cross-sectional survey, only respondents who came for ANC visit were interviewed, thereby excluding those who did not visit the health centers. Moreover, this study was conducted at health centers in a major town or urban administration, which could have overstated the accessibility of women to mobile phones and their willingness to receive the SMS text message–based mHealth intervention. In addition, the survey was interviewer-administered, and even if we used neutral interviewers, there might be an interviewer and social desirability bias that could have made more participants to respond in the affirmative. These limitations have to be considered when generalizing these results.

### Conclusions

A high proportion of respondents attending ANC clinics in a resource-poor urban setting of Ethiopia have mobile phone access and are willing to receive SMS text message–based mHealth interventions. Thus, mobile phone–based interventions to improve maternal health should be tried and explored further. Moreover, age, educational status, and frequency of the mobile phone use are significant factors associated with the willingness.

## Abbreviations

ANC: antenatal care
AOR: adjusted odds ratio
ART: antiretroviral therapy
SIM: subscriber identity module or subscriber identification module
SMS: short message service

## References

[1] UNFPA (2016). World Bank Group and the United Nations Population Division: Trends in Maternal Mortality to 2015. World Bank Group and the United Nations Population Division: Trends in Maternal Mortality to 2015.

[2] WHO (2016). WHO Guidelines Approved by the Guidelines Review Committee:WHO Recommendations on Antenatal Care for a Positive Pregnancy Experience. WHO Guidelines Approved by the Guidelines Review Committee:WHO Recommendations on Antenatal Care for a Positive Pregnancy Experience.

[3] Alkema Leontine, Chou Doris, Hogan Daniel, Zhang Sanqian, Moller Ann-Beth, Gemmill Alison, Fat Doris Ma, Boerma Ties, Temmerman Marleen, Mathers Colin, Say Lale, United Nations Maternal Mortality Estimation Inter-Agency Group collaboratorstechnical advisory group (2016). Global, regional, and national levels and trends in maternal mortality between 1990 and 2015, with scenario-based projections to 2030: a systematic analysis by the UN Maternal Mortality Estimation Inter-Agency Group. Lancet.

[4] Abalos E, Chamillard M, Diaz V, Tuncalp Ӧ, Gülmezoglu A M (2016). Antenatal care for healthy pregnant women: a mapping of interventions from existing guidelines to inform the development of new WHO guidance on antenatal care. BJOG.

[5] Central Statistical Agency (CSA) (2016). Ethiopia Demographic and Health Survey. Ethiopia Demographic and Health Survey.

[6] Campbell James, Admasu Kesetebirhan, Soucat Agnes, Tlou Sheila (2015). Maximizing the impact of community-based practitioners in the quest for universal health coverage. Bull World Health Organ.

[7] Vodopivec-Jamsek Vlasta, de Jongh Thyra, Gurol-Urganci Ipek, Atun Rifat, Car Josip (2012). Mobile phone messaging for preventive health care. Cochrane Database Syst Rev.

[8] Gibson Dustin G, Kagucia E Wangeci, Ochieng Benard, Hariharan Nisha, Obor David, Moulton Lawrence H, Winch Peter J, Levine Orin S, Odhiambo Frank, O'Brien Katherine L, Feikin Daniel R (2016). The Mobile Solutions for Immunization (M-SIMU) Trial: A Protocol for a Cluster Randomized Controlled Trial That Assesses the Impact of Mobile Phone Delivered Reminders and Travel Subsidies to Improve Childhood Immunization Coverage Rates and Timeliness in Western Kenya. JMIR Res Protoc.

[9] Jennings Larissa, Omoni Adetayo, Akerele Akunle, Ibrahim Yisa, Ekanem Ekpenyong (2015). Disparities in mobile phone access and maternal health service utilization in Nigeria: a population-based survey. Int J Med Inform.

[10] Ngabo Fidele, Nguimfack Judith, Nwaigwe Friday, Mugeni Catherine, Muhoza Denis, Wilson David R, Kalach John, Gakuba Richard, Karema Corrine, Binagwaho Agnes (2012). Designing and Implementing an Innovative SMS-based alert system (RapidSMS-MCH) to monitor pregnancy and reduce maternal and child deaths in Rwanda. Pan Afr Med J.

[11] Lund S, Hemed M, Nielsen B B, Said A, Said K, Makungu M H, Rasch V (2012). Mobile phones as a health communication tool to improve skilled attendance at delivery in Zanzibar: a cluster-randomised controlled trial. BJOG.

[12] Tilahun Binyam, Smillie Kirsten, Bardosh Kevin Louis, Murray Melanie, Fitzgerald Mark, Cook Victoria, Poureslami Iraj, Forrest Jamie, Lester Richard (2018). Identifying Barriers and Facilitators of 13 mHealth Projects in North America and Africa: Protocol for a 5-Year Implementation Science Study. JMIR Res Protoc.

[13] Lau Yan Kwan, Cassidy Tali, Hacking Damian, Brittain Kirsty, Haricharan Hanne Jensen, Heap Marion (2014). Antenatal health promotion via short message service at a Midwife Obstetrics Unit in South Africa: a mixed methods study. BMC Pregnancy Childbirth.

[14] Bastawrous Andrew, Armstrong Matthew J (2013). Mobile health use in low- and high-income countries: an overview of the peer-reviewed literature. J R Soc Med.

[15] Balakrishnan Ramkrishnan, Gopichandran Vijayaprasad, Chaturvedi Sharadprakash, Chatterjee Rahul, Mahapatra Tanmay, Chaudhuri Indrajit (2016). Continuum of Care Services for Maternal and Child Health using mobile technology - a health system strengthening strategy in low and middle income countries. BMC Med Inform Decis Mak.

[16] Evans William, Nielsen Peter E, Szekely Daniel R, Bihm Jasmine W, Murray Elizabeth A, Snider Jeremy, Abroms Lorien C (2015). Dose-response effects of the text4baby mobile health program: randomized controlled trial. JMIR Mhealth Uhealth.

[17] Datta Shib Sekhar, Ranganathan Pandiyan, Sivakumar Krithiga S (2014). A study to assess the feasibility of Text Messaging Service in delivering maternal and child healthcare messages in a rural area of Tamil Nadu, India. Australas Med J.

[18] Poorman Elisabeth, Gazmararian Julie, Parker Ruth M, Yang Baiyu, Elon Lisa (2015). Use of text messaging for maternal and infant health: a systematic review of the literature. Matern Child Health J.

[19] Cormick Gabriela, Kim Natalie A, Rodgers Ashlei, Gibbons Luz, Buekens Pierre M, Belizán José M, Althabe Fernando (2012). Interest of pregnant women in the use of SMS (short message service) text messages for the improvement of perinatal and postnatal care. Reprod Health.

[20] Alwan Kalid, Awoke Tadesse, Tilahun Binyam (2015). Knowledge and Utilization of Computers Among Health Professionals in a Developing Country: A Cross-Sectional Study. JMIR Hum Factors.

[21] van Heerden Alastair, Norris Shane, Tollman Stephen, Richter Linda, Rotheram-Borus Mary Jane (2013). Collecting maternal health information from HIV-positive pregnant women using mobile phone-assisted face-to-face interviews in Southern Africa. J Med Internet Res.

[22] ITU (2016). Measuring the Information Society Report. Measuring the Information Society Report.

[23] Kazi Abdul Momin, Carmichael Jason-Louis, Hapanna Galgallo Waqo, Wangoo Patrick Gikaria, Karanja Sarah, Wanyama Denis, Muhula Samuel Opondo, Kyomuhangi Lennie Bazira, Loolpapit Mores, Wangalwa Gilbert Bwire, Kinagwi Koki, Lester Richard Todd (2017). Assessing Mobile Phone Access and Perceptions for Texting-Based mHealth Interventions Among Expectant Mothers and Child Caregivers in Remote Regions of Northern Kenya: A Survey-Based Descriptive Study. JMIR Public Health Surveill.

[24] Kebede Mihiretu, Zeleke Atinkut, Asemahagn Mulusew, Fritz Fleur (2015). Willingness to receive text message medication reminders among patients on antiretroviral treatment in North West Ethiopia: A cross-sectional study. BMC Med Inform Decis Mak.

[25] Nachega Jean B, Skinner Donald, Jennings Larissa, Magidson Jessica F, Altice Frederick L, Burke Jessica G, Lester Richard T, Uthman Olalekan A, Knowlton Amy R, Cotton Mark F, Anderson Jean R, Theron Gerhard B (2016). Acceptability and feasibility of mHealth and community-based directly observed antiretroviral therapy to prevent mother-to-child HIV transmission in South African pregnant women under Option B+: an exploratory study. Patient Prefer Adherence.

[26] Zurovac Dejan, Otieno Gabriel, Kigen Samuel, Mbithi Agneta M, Muturi Alex, Snow Robert W, Nyandigisi Andrew (2013). Ownership and use of mobile phones among health workers, caregivers of sick children and adult patients in Kenya: cross-sectional national survey. Global Health.

[27] Balogun M R, Sekoni A O, Campbell P C (2012). Access to information technology and willingness to receive text message reminders for childhood immunisation among mothers attending a tertiary facility in Lagos, Nigeria. Access to information technology and willingness to receive text message reminders for childhood immunisation among mothers attending a tertiary facility in Lagos, Nigeria.

